# Left Internal Iliac Artery Aneurysm Presenting With Nephrostomy-Site and Rectal Bleeding From an Arterio-Ureteral Fistula in a Patient With Rectovesical Fistula After Colorectal Cancer Therapy: A Report of a Rare Case

**DOI:** 10.7759/cureus.96875

**Published:** 2025-11-14

**Authors:** Muhammad Ashif Khan Abdullah, A B Azharul Islam

**Affiliations:** 1 Urology, Countess of Chester Hospital NHS Foundation Trust, Chester, GBR

**Keywords:** arterio-ureteral fistula, endovascular embolisation, haematuria, internal iliac artery aneurysm, post-colorectal cancer complications

## Abstract

Internal iliac artery aneurysms (IIAAs) are rare vascular abnormalities, while arterio-ureteral fistulas (AUFs) are uncommon but potentially fatal causes of hematuria. Both conditions are typically associated with prior pelvic surgery, radiotherapy, or long-term ureteric stenting. We describe the case of a 75-year-old man with a history of colorectal carcinoma managed with surgery and adjuvant pelvic radiotherapy, complicated by a rectovesical fistula and bilateral nephrostomies for obstructive uropathy. The patient presented with intermittent bleeding from the left nephrostomy and per rectum. A CT angiography demonstrated a left internal iliac artery aneurysm arising from the anterior division, in proximity to the distal left ureteric stent, raising suspicion of an arterio‑ureteral fistula. Endovascular coil embolization of the aneurysm was performed, resulting in complete resolution of bleeding. Follow-up CT angiography performed one month after the procedure confirmed complete exclusion of the aneurysm and absence of recurrent bleeding. This case illustrates a rare presentation of an internal iliac artery aneurysm with an arterio-ureteral fistula in the setting of a rectovesical fistula after colorectal cancer treatment, emphasizing the importance of early recognition and prompt endovascular management in complex post-oncologic pelvic bleeding.

## Introduction

Isolated internal iliac artery aneurysms (IIAAs) are uncommon, accounting for less than 0.4% of all aneurysms and approximately 2% of aortoiliac aneurysmal disease. Their deep pelvic location often results in late presentation, and rupture carries a mortality rate of up to 50% [1]. With the evolution of endovascular therapy, management outcomes have improved significantly, offering a less invasive and safer alternative to open surgical repair, particularly in patients with previous pelvic surgery or radiotherapy [2].

Arterio-ureteral fistulas (AUFs) represent another rare but life-threatening vascular complication, increasingly recognized due to the widespread use of chronic indwelling ureteral stents and pelvic oncologic surgery [3,4]. They usually occur where the ureter crosses the iliac arteries, with the pathogenesis involving chronic frictional trauma, fibrosis, and radiation-induced arterial fragility [4,5]. Clinically, AUFs manifest as intermittent or massive hematuria and are often difficult to diagnose because of episodic bleeding and non-specific imaging findings [5,6].

In patients with prior pelvic malignancy, radiation, or chronic urinary diversion, vascular injuries can coexist with fistulous tracts involving adjacent organs. The simultaneous occurrence of an internal iliac artery aneurysm and an arterio-ureteral fistula is exceptionally rare. This report describes a patient with rectal and nephrostomy-site bleeding secondary to a left internal iliac artery aneurysm eroding into a chronically stented ureter. The case highlights the importance of clinical suspicion and the role of endovascular techniques in managing complex post-oncologic pelvic hemorrhage.

## Case presentation

Clinical history

A 75-year-old male with a history of rectosigmoid adenocarcinoma underwent Hartmann’s procedure in 2015, followed by adjuvant chemoradiotherapy. He subsequently developed a chronic rectovesical fistula managed conservatively. Long-term bilateral nephrostomies were required for ureteric obstruction, and a left metallic ureteric stent was placed in 2021 for persistent obstruction.

The patient presented with intermittent bleeding from the left nephrostomy site and per rectum, accompanied by mild lower abdominal discomfort. He was hemodynamically stable but demonstrated a progressive hemoglobin drop (12.0 → 9.5 g/dL). The stoma was healthy, the right nephrostomy was clear, and the left nephrostomy drained blood-tinged urine.

Investigations

**Figure 1:**
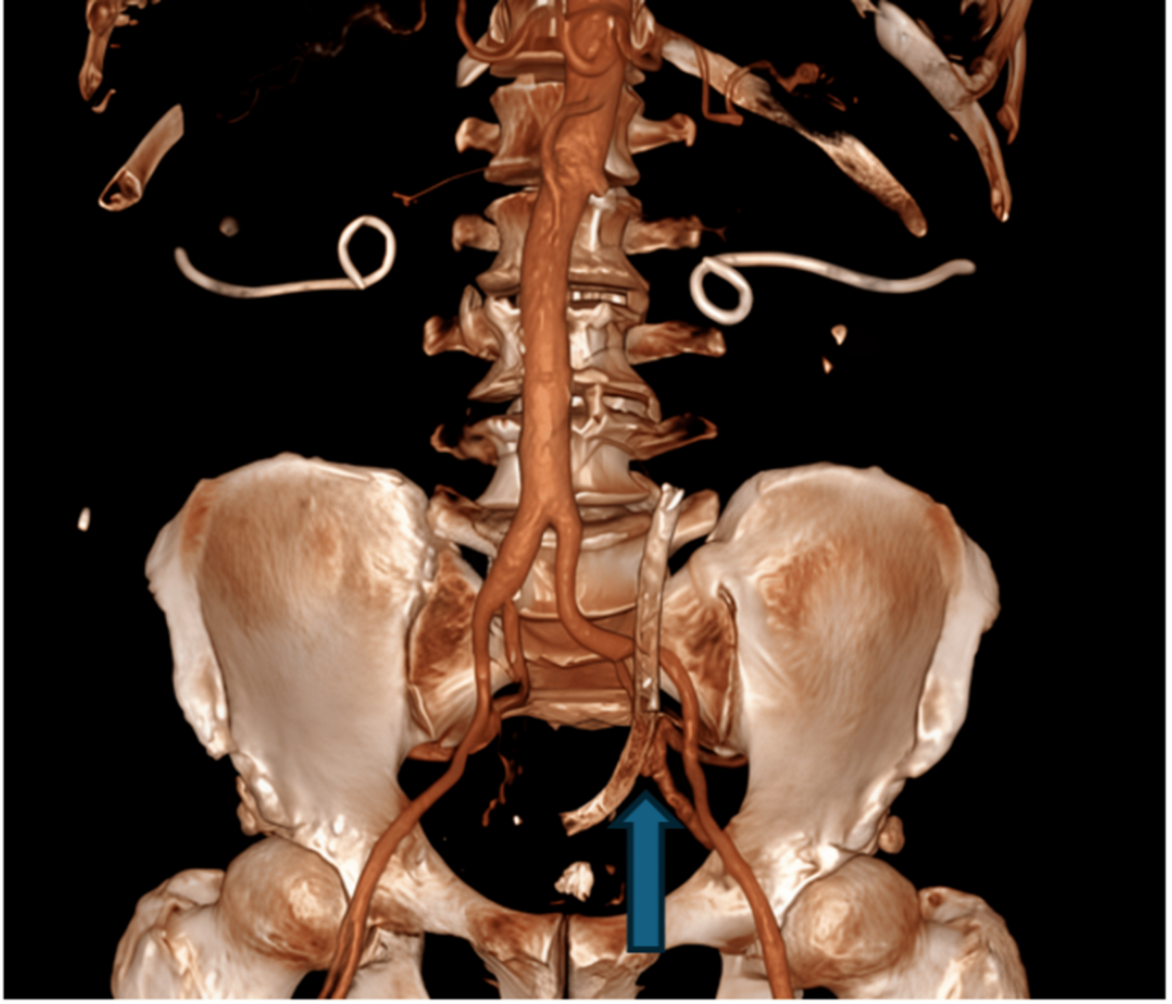
3D CT angiography showing left internal iliac artery aneurysm adjacent to ureteric metallic stent (blue arrow)

**Figure 2:**
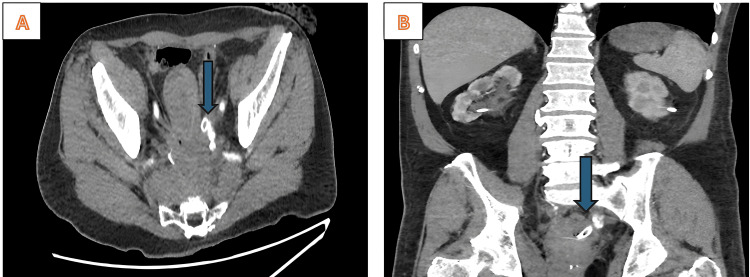
CT angiography demonstrating left internal iliac artery aneurysm adjacent to a ureteric stent A: Axial image showing a saccular aneurysm of the left internal iliac artery (arrow) closely abutting the distal ureteric stent; B: Coronal reconstruction confirms the aneurysm’s proximity to the distal left ureter (arrow), consistent with an arterio-ureteral fistula.

Laboratory tests showed normal coagulation and renal function. Urinalysis confirmed hematuria without infection. A CT angiography of the abdomen and pelvis revealed a saccular aneurysm arising from the anterior division of the left internal iliac artery, closely abutting the distal left ureteric stent. No active extravasation was visible, but the anatomic proximity raised suspicion of an arterio-ureteral fistula. Figure 1 features the 3D CTA revealing a left internal iliac artery aneurysm adjacent to a ureteric metallic stent. Figure 2 shows the CT angiography of the left internal iliac artery aneurysm adjacent to a ureteric stent.

Management

**Figure 3:**
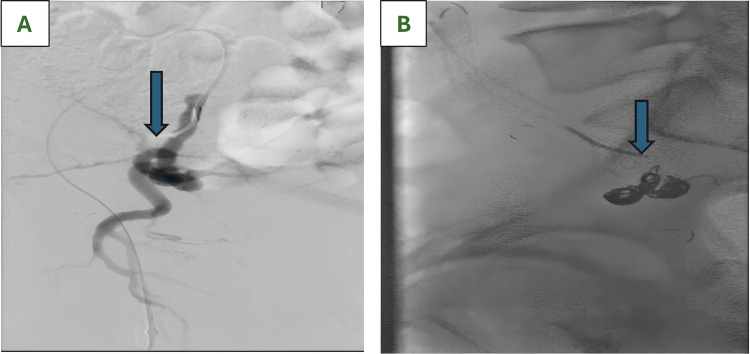
Digital subtraction angiography of the left internal iliac artery aneurysm with arterio-ureteral fistula A: Selective angiogram showing a left internal iliac artery aneurysm (arrow); B: Post-embolization image demonstrating complete aneurysm exclusion by coil embolization (arrow)

An urgent angiogram was performed by interventional radiology. Selective catheterization of the left internal iliac artery demonstrated an aneurysm arising from the anterior division in proximity to the left ureteric stent, with sluggish filling but no frank rupture. Although no active extravasation was observed, the close anatomical relationship suggested a probable fistulous communication, which was managed by endovascular exclusion. Endovascular coil embolization of the aneurysm sac was performed using multiple detachable Ruby coils (8 cm × 25 cm, 6 cm × 30 cm, and a 45 cm packing coil), resulting in dense packing and complete exclusion of the lesion. Adjacent internal iliac branches were also embolized, and the posterior division remained patent. There were no immediate procedural complications (Figure 3). Post-procedure, bleeding from the nephrostomy and rectum ceased. The patient remained haemodynamically stable.

Outcome and follow-up

At the one-month follow-up, the patient remained clinically stable with no recurrence of hematuria or rectal bleeding. Cystoscopy and bladder washout revealed residual clots and the pre-existing fistulous opening on the posterior bladder wall, without evidence of ongoing hemorrhage. At three months, the patient continued to do well with functioning nephrostomies and no signs of recurrent bleeding.

## Discussion

The IIAAs are the least frequent of all intra-abdominal aneurysms but carry a high risk of rupture and hemorrhage when left untreated [1]. Most are atherosclerotic, but prior pelvic surgery, infection, or radiotherapy can contribute to aneurysm formation by damaging the arterial wall [1,2]. Endovascular techniques, including coil embolization or stent-graft placement, are now considered first-line management for IIAAs due to their lower morbidity and shorter recovery times compared with open repair [2].

Arterio-ureteral fistulas, while rare, are increasingly identified in patients with previous pelvic malignancy, radiation therapy, or chronic ureteral stents. Kamphorst et al. [3] analyzed 445 cases of arterio‑ureteral fistula and found that more than 80% were associated with prior pelvic surgery or radiation, underscoring its predominantly iatrogenic origin. The external and common iliac arteries are most commonly affected, while involvement of the internal iliac artery is distinctly unusual [4].

Diagnosing AUF remains challenging because bleeding is often intermittent, and imaging findings are inconclusive. The CT angiography is the usual first-line investigation, but may not always demonstrate the fistula or active extravasation [5]. Selective or provocative angiography, sometimes performed with temporary stent manipulation, has been shown to improve diagnostic accuracy and remains the reference standard when suspicion is high [6].

Endovascular management has become the treatment of choice in hemodynamically stable patients. It provides immediate hemostasis and allows exclusion of the involved arterial segment with minimal surgical trauma. Subiela et al. [7] reported high technical success and durable outcomes in a systematic review and single-center experience, while more recent reports confirm that endovascular repair is safe even in complex or irradiated pelvic anatomy [8].

In the present case, long-term ureteric stenting and radiation-induced tissue changes likely contributed to aneurysm wall erosion and formation of an arterio-ureteral fistula. The coexistence of a rectovesical fistula may have facilitated the passage of arterial blood into the rectum, potentially explaining the simultaneous nephrostomy-site and rectal bleeding observed in this case. The successful coil embolization of the internal iliac artery aneurysm achieved rapid and sustained control of hemorrhage, consistent with outcomes reported in the literature [7,8].

A multidisciplinary approach involving vascular surgery, urology, and interventional radiology is critical for optimal management. Long-term imaging follow-up is essential to ensure aneurysm exclusion, assess for recurrent bleeding, and detect potential secondary fistula formation in this high-risk patient population.

## Conclusions

We describe a rare case of a left internal iliac artery aneurysm producing an arterio-ureteral fistula in a patient with a chronic rectovesical fistula after colorectal cancer therapy. The patient presented with concurrent nephrostomy and rectal bleeding, successfully managed by endovascular embolization. This case emphasizes the need to maintain a high index of suspicion for vascular-urinary communication in patients with prior pelvic malignancy, radiotherapy, or chronic ureteric stents, and to promptly employ CT angiography or selective angiography when unexplained hematuria occurs.
